# Temporal trends of cancer incidence rates for the most frequent cancer sites in Cyprus (2004–2017)

**DOI:** 10.1002/cnr2.2000

**Published:** 2024-06-12

**Authors:** Anastasia Spartiati, Anna Demetriou, V. Scoutellas, Costas A. Christophi, Konstantinos C. Makris

**Affiliations:** ^1^ Cyprus International Institute for Environmental and Public Health, School of Health Sciences Cyprus University of Technology Limassol Cyprus; ^2^ Cancer Registry Ministry of Health Nicosia Cyprus

**Keywords:** annual percentage change, breast, cancer, joinpoint, thyroid

## Abstract

**Background:**

Cancer is one of the leading causes of morbidity and mortality, worldwide. Little information is available for the temporal trends of cancer in the Mediterranean region, including Cyprus.

**Aims:**

We aimed to analyze cancer incidence trends overall and by sex for the period 2004–2017 regarding the five most common cancer sites for the population of Cyprus.

**Methods and results:**

Data were obtained from the nationwide cancer registry dataset that included 27 017 total cancer cases in Cyprus (2004–2017). We estimated the crude, sex‐, and age‐specific, as well as age‐standardized (ASR) cancer incidence rates and we analyzed the time trends of ASR using the joinpoint regression program. For the general population (0–85+ years of age), the most common cancer sites in descending order, were breast, prostate, lung, colorectal, and thyroid cancer. During the study period, breast and thyroid cancer ASR presented a significant (*p* < .05) increasing temporal trend. Lung cancer ASRs seemed to stabilize (no increase or decrease) during the more recent years (2009 onwards) for both sexes; a similar pattern was observed for colorectal cancer in males. The ASRs of prostate cancer in men were in steady decline from 2012 onwards and the same was observed for the female ASRs of colorectal cancer from 2007 onwards. The colorectal cancer ASR temporal patterns overall, during the whole study period appeared unchanged.

**Conclusion:**

This temporal analysis would feed into cancer surveillance and control programs that focus on prevention, early detection, and treatment, particularly for cancer sites of higher mortality rates or those with temporally increasing trends.

## 1 INTRODUCTION

Cancer is one of the leading chronic diseases and causes of morbidity and mortality worldwide, with approximately 19 million new cases and 10 million deaths in 2020.^1^, ^2^, ^3^ Cancer is the second cause of death.^4^ The European population is about one tenth of the world's population, however, it reports almost 25% of all cancer cases, globally.^5^ Moreover, in Europe, cancer is the disease with the highest mortality rates in people <65 years old compared to other diseases.^5^ In 2020, there were an estimated 2.6 million new cancer cases and approximately 1.3 million cancer deaths in the European Union.^5^ From 1995 to 2008, it has been reported that the numbers of new cases and deaths have increased by 50% and by 20%, respectively.^6^ The number of cancer cases in Europe is projected to increase to 4.75 million cases and 2.55 million deaths by 2040 primarily because of population aging and growth.^7^

The Republic of Cyprus is part of the European Union and it is an island located in the Eastern Mediterranean region. According to the latest Census in 2021, the population of the Republic of Cyprus was 918 000 with 51.4% women.^8^ In 2020, the leading causes of death in Cyprus were cardiovascular diseases, cancer and diabetes for both sexes, while cancer mortality rates have remained stable, since 2004.^9^ About one in four deaths in Cyprus are from cancer, while lung cancer is the most frequent cause of cancer deaths.^9^

Since 1994, Cyprus joined the Middle East Cancer Society, while in 1996, it also joined the Middle East Cancer Consortium (MECC). In 1998, MECC established the Cancer Registry Project, through which Cyprus created its Cyprus Cancer Registry (CyCR),^10^ although it was initially established in 1990. Initially, the CyCR covered ~88% of the cases, with that number rising to 95% by 2008.^11^ The latest cancer data for Cyprus come from CyCR in 2020 that reported 4078 new cancer cases.^12^ Among Cypriot males, the most common cancer sites were prostate followed by lung and colorectal, while in females the most frequent cancer sites were breast, followed by thyroid and colorectal.^12^ In the period 1998–2020 for males, there were 9197 prostate cancer, 4666 lung, and 4147 colorectal cancer cases; during the same period for females, there were 11 059 breast cancer, 3343 thyroid, and 3113 colorectal cancer cases.^12^ In 2020, the frequency of the top cancer sites remained the same^12^ with that of previous years for both sexes. According to the Cyprus health profile in 2021, cancer mortality in men was higher among those diagnosed with lung cancer, followed by prostate and colorectal cancer, while for women, breast, followed by lung and colorectum cancer had the highest mortality rates, in descending order.^9^

In Cyprus, cancer epidemiological data are frequently reported by the Ministry of Health website,^13^ but the publication of cancer temporal trends has not been routinely practiced. The knowledge of temporal trends of cancer sites in a population is of utmost importance in informing population programs for cancer prevention. The objective of this study was to analyze cancer incidence trends by sex and calendar year for the period of 2004–2017 for the five most common cancers of the population of Cyprus. This was accomplished using data from the CyCR database.

## 2 METHODOLOGY

### 2.1 Study area and population

The study was implemented within the government‐controlled territories of the Republic of Cyprus. Thus, the study population were Cypriots of all ages, including those who had declared Cyprus as the country of permanent residence during the study period 2004–2017. The Cyprus National Bioethics Committee approved the study protocol (EEBΚ/ΕΠ/2019/01/171), while the Cancer Registry Unit of the Ministry of Health provided the relevant data.

### 2.2 Data sources

Pseudoanonymized data was obtained from the CyCR, which is a population‐based cancer registry administrated by the Cyprus Ministry of Health using the coding and staging system of the MECC and the U.S. National Cancer Institute.^14^ The CyCR was established in 1996 after the initiative of the MECC. The registry is part of the Health Monitoring Unit (HMU) of the Ministry of Health. The Permanent Secretary of the Ministry of Health has the administrative responsibility for the CyCR. The CyCR is a member of the IACR (International Agency for Cancer Registries) and ENCR (European Network of Cancer Registries) and periodically reports cancer data to these agencies. At the CyCR, specially trained people with experience of more than 18 years, code the data retrieved from notifications of cancer in accordance with International, and European guidelines for cancer registration. ICDO‐3, ICD‐10, SEER Summary Staging and TNM (Tumor, Node, and Metastases) are some of the coding guidelines that were used.

Information from all public and private hospitals, as well as clinics, within the Republic of Cyprus between 2004 and 2017 was included. Population and demographic data were obtained from the Census population and housing survey (Cyprus Statistical Service, 2011),^15^ while the end of year annual population estimates were obtained from the Statistical Service of the Republic of Cyprus (https://www.cystat.gov.cy/el/PublicationList?s=46).

### 2.3 Variables

For each registered cancer case, sex and age were recorded, including the first diagnosis date, and the type of cancer. Covariates, like smoking history, stage at diagnosis and histology type were also recorded. The five most popular cancer types in Cyprus for both sexes combined were used, i.e., breast, prostate, colorectal, lung and thyroid cancer; cancer cases were stratified by sex and by cancer type.

Cancer types were classified based on the International Classification of Diseases ICD‐10 (C18‐C20 for colorectal, C34 for lung, C50 for breast, C61 for prostate, and C73 for thyroid). Cases were also classified according to ICDO3 histological codes and four groups were created, i.e., neoplasm, carcinoma, sarcoma and others. Cancer stages at diagnosis included in situ, localized, regional by direct extension, regional by lymph nodes, regional by both direct extension and lymph nodes, regional not otherwise specified, distant, and unknown/undetermined. There were 18 age groups (0–4, 5–9, 10–14, 15–19, 20–24, 25–29, 30–34, 35–36, 40–44, 45–49, 50–54, 55–59, 60–64, 65–69, 70–74, 75–79, 80–84, and 85+). Smoking status was categorized in four groups (current, former, never smoker and unknown).

### 2.4 Statistical analysis

Crude rates were defined as the total number of events divided by the mid‐year total population of the selected geography and multiplied by 100 000 for the population at risk. For each cancer type, the annual crude rate was calculated separately as the number of cases (R) divided by the expected person‐years in that year (N) and multiplied by 100 000. Similarly, to calculate the age‐specific rates, the number of cases in each age category was divided by the expected person‐years in that age category and multiplied by 100 000.^16^

Age‐standardized rates (ASR) were also calculated to provide direct standardization for age differences between populations.^17^ These are expressed as cases per 100 000 inhabitants and they report the cancer incidence that would be expected in the region, if the region had the age structure of the standard population. The standard population for the calculation of the ASRs in this study was the European Standard population.^18^ The above‐mentioned metrics were applied to each age‐group and sex to study cancer incidence rate differences by histology type and smoking status, performed by age‐group and sex. The ASRs were used as input for the temporal trend analysis. The analysis was performed using R Studio (version 4.2.0).

### 2.5 Temporal Trend Analysis

To evaluate cancer incidence rate trends between 2004 and 2017, a time‐series analysis was implemented using ASRs. The temporal trend analysis was performed using the Joinpoint Regression Program software (version 4.9.1.0, National Cancer Institute, USA) that was originally developed for the analysis of data from the U.S. NIH Surveillance Epidemiology and End Results Program (SEER).^19^ This program identifies the optimum model fit of several years of data, utilizing an algorithm that tests whether a multi‐segment line is significantly better fit than a straight or less segment line using the so‐called “joinpoints.” Each joinpoint denotes a statistically significant change in cancer trend, using a test of significance that is based on a Monte Carlo Permutation test to locate the best fit for each joinpoint line.^20^ In this analysis, because of the relatively small study period, a maximum of two joinpoints was allowed in each model.^21^ The annual percentage change (APC) of the ASR was tested to determine whether a difference exists from the null hypothesis of no change. In the final model, each joinpoint informs a statistically significant change in trends (increase or decrease).^22^ The terms increase and decrease were used when the slope of the trend (annual percentage change, APC or average annual percentage change, AAPC) were statistically significant using two‐sided tests (*p* < .05) (using a *t*‐test for both the APC and AAPC when it lied entirely within the last joinpoint segment, and a *z*‐test when the AAPC extended beyond the last joinpoint segment). The APC was calculated for rates between trend‐change points, and the AAPC for the whole study period. Based on the suggested software model fits, the APCs and the AAPC were calculated by fitting log‐linear trendlines to the time‐series datasets, assuming constant variance.^19^, ^22^

## 3 RESULTS

### 3.1 Cancer data quality and descriptive epidemiology

Quality assurance and quality control indicators are routinely used by the Cyprus Cancer Registry as part of the annual cancer data provided for each cancer site. The quality of cancer registry information is annually assessed by the following internationally harmonized standard parameters: the percentage of microscopically (histologically or cytologically) confirmed cases and the percentage of cancer cases registered on the basis of death certificates only (Table S1).

During 2004–2017, there were 27 017 registered cancer cases in the general population of Cyprus, of which 50.5% (*n* = 13 643) of them were males and 49.5% (*n* = 13 374) were females (Table 1). The mean age at first cancer diagnosis was 64 years (69 years for males and 59 years for females). In that same period, the five most common cancer types overall, in descending order, were breast (7487 cases), prostate (6203 cases), colorectal (4961 cases), lung (3971 cases), and thyroid (2974 cases) cancer. Among men, the five most common cancers, in descending order, were prostate (6203 cases), lung (3080 cases), colorectal (2855 cases), thyroid (580 cases), and lymphocytic leukemia (451 cases), while in women, these were breast (7417 cases), thyroid (2394 cases), colorectal (2106 cases), lung (891 cases), and lymphocytic leukemia (276 cases).

**TABLE 1:** The overall stratified crude incidence rates (cIR) and age standardized rates (ASR) per 100 000 for the five most common cancer sites (breast, prostate, lung, colorectal, and thyroid) in Cyprus during 2017, total and by age group.

	Overall			Men			Women		
	*N*	cIR	ASR	*N*	cIR	ASR	*N*	cIR	ASR
Colorectal cancer									
Total	352	37	42	207	44	53	144	36	34
0–19	1	0	0	0	0	0	1	10	2
20–64	119	20	12	63	22	13	55	18	11
65+	232	153	30	144	205	40	88	108	21
Lung cancer									
Total	345	36	41	267	57	68	78	20	17
0–19	0	0	0	0	0	0	0	0	0
20–64	108	18	11	78	27	16	30	10	6
65+	237	156	30	187	270	53	48	59	11
Breast cancer									
Total	620	65	69	3	1	1	617	155	131
0–19	0	0	0	0	0	0	0	0	0
20–64	367	62	36	1	0	0	366	120	71
65+	253	167	32	2	3	1	251	307	60
Prostate cancer									
Total				478	102	125			
0–19				0	0	0			
20–64				110	38	22			
65+				368	525	102			
Thyroid cancer									
Total	357	37	37	64	14	14	293	74	68
0–19	5	2	1	0	0	0	5	49	10
20–64	292	49	29	46	16	9	246	81	48
65+	60	37	8	18	26	5	42	51	10

The highest annual crude rate (cIR) and ASR in 2017 for Cyprus among the five most common cancer sites for both sexes was for breast cancer, 65 and 69 per 100 000, respectively, however, high rates were observed for prostate cancer (102 and 125 per 100 000, respectively). Colorectal and lung cancer had similar cIR and ASR; for colorectal cancer the cIR and ASR was 37 and 42 per 100 000, and 36 and 41 per 100 000 respectively, for lung cancer; the thyroid cancer cIR and ASR were 37 and 37 per 100 000. Among the three age groups, an increase from the adult group (20–64 years of age) to the elderly group (65+) in ASRs was observed for all cancer sites, except for breast cancer, which had similar ASRs between the adult and the elderly groups (Table 1). Sex differences in ASRs in 2017 was found for the colorectal cancer and lung cancer sites (higher for men).

The majority of registered cancer stages were those of stage 1, local only (35.2%), followed by stage 7, distant (16.5%), then Stage 3, regionally affected lymph nodes only (9.9%), stage 2, regional with direct extensions only (9.6%), and stage 4, regional with direct and through the lymph nodes extension (7.3%) (Table S1). Almost half of the cases (44.5%, *n* = 12 016) had unknown smoking status, however, among current smokers during cancer diagnosis (*n* = 4241, 15.7%) the ASR was higher in males than females (annual ASR of 903 and 347 per 100 000 persons, respectively). The ASR of former smokers (*n* = 3205, 11.9%) was much higher in males than females (annual ASR of 983 vs. 98 per 100 000 persons, respectively), while among those who never smoke (*n* = 7555, 28%) females had a higher annual ASR than males. Of all cancer cases, carcinomas (88.4%) exhibited the highest annual cIR and ASR (2842 and 3592, per 100 000 people), while there were much fewer neoplasms (11.5%) and minor sarcomas or other histological types (Table S1).

### 3.2 Temporal trends

**FIGURE 1:**
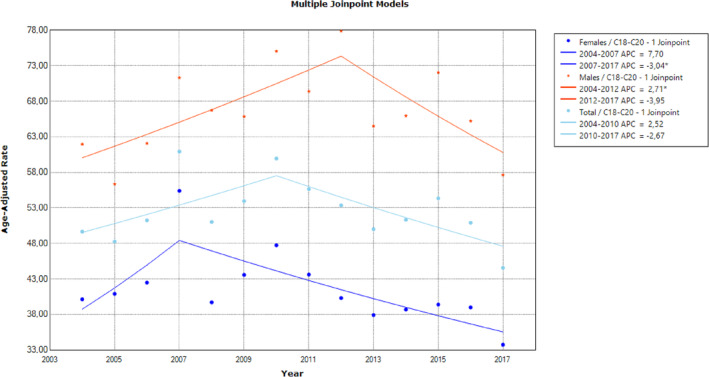
Age‐standardized colorectal cancer incidence rates per 100 000 persons per year in Cyprus between 2004 and 2017, for the whole population and by sex (* denotes significant time trend).

**FIGURE 2:**
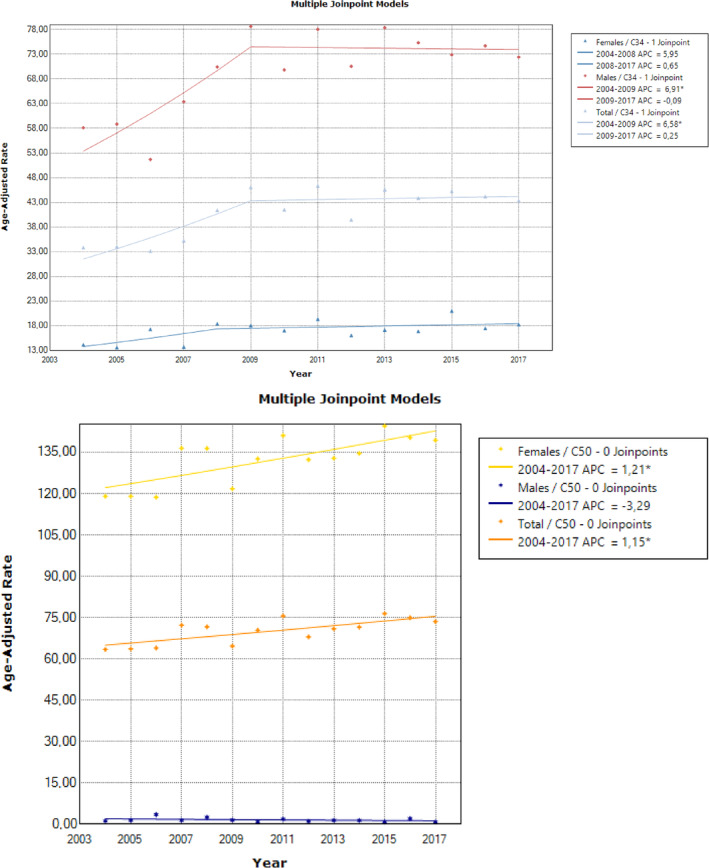
(A). Age‐standardized lung cancer incidence rates per 100 000 persons per year in Cyprus between 2004 and 2017, for the whole population and by sex (* denotes significant trend). (B). Age‐standardized breast cancer incidence rates per 100 000 persons per year in Cyprus between 2004 and 2017, for the whole population and by sex (* denotes significant trend).

**FIGURE 3:**
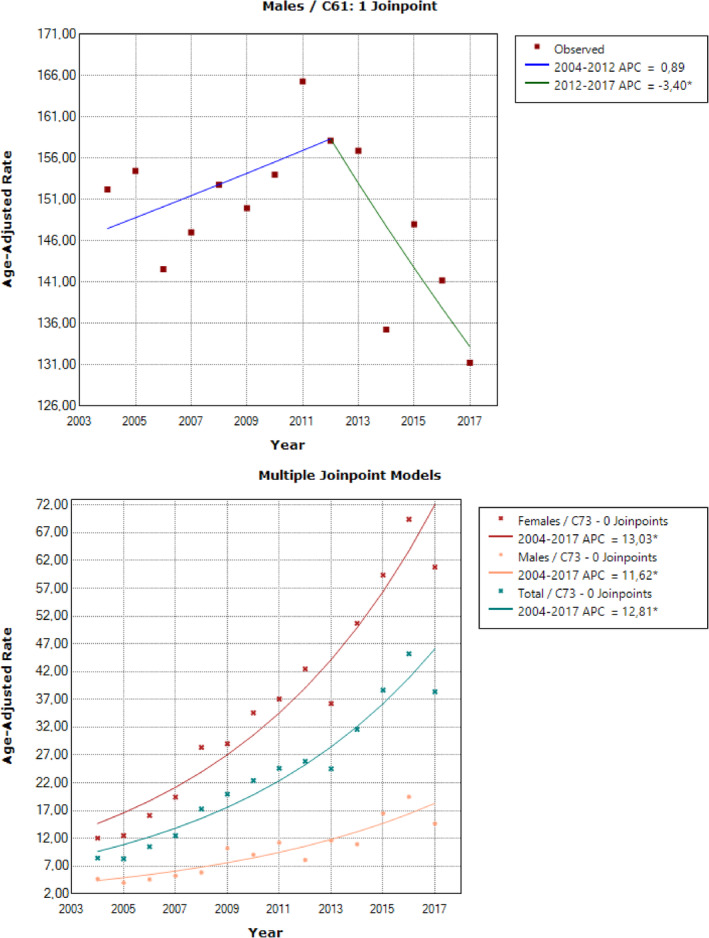
(A). Age‐standardized prostate cancer incidence rates per 100 000 persons per year in Cyprus between 2004 and 2017, for the whole population and by sex (* denotes significant trend). (B). Age‐standardized thyroid cancer incidence rates per 100 000 persons per year in Cyprus between 2004 and 2017, for the whole population and by sex (* denotes significant trend).

The results of the joinpoint analysis for the five main cancer types in the general population of Cyprus are visualized in Figures 1, 2, 3 and in Table S2. It was observed that colorectal cancer ASR fluctuated overall during the 2004–2017; non‐significant upwards (APC = 2.5%; 95% CI: −1.5, 6.7, *p* = .195) and downwards (APC = −2.7%, 95% CI: −5.4, 0.1, *p* = .06) changes in APCs were observed until 2010, and after 2010, respectively (Figure 1). The corresponding average annual percentage change (AAPC) was −0.3 (95% CI: −2.4, 1.8, *p* = .773). Males exhibited an increasing temporal pattern from 2004 to 2012 (APC = 2.7%, 95% CI: 0.1, 5.4, *p* < .046), but the downward change from 2012 onwards was not statistically significant (*p* > .05) (APC = −3.90%, 95% CI: −8.6, 0.9, *p* > .097). Females had lower ASR colorectal cancer incidence rates than males and their ASR showed a significant annual decrease from 2007 onwards (APC = − 3.00%, 95% CI: −4.6, −1.4, *p* = .002).

Lung cancer ASRs for the whole population showed an annual increase until 2009 (APC = 6.6%, 95% CI: 1.6, 11.8, *p* = .014), but they stabilized over the remaining period with an overall AAPC of 2.6% (95% CI: 0.7, 4.6, *p* = .007) (Figure 2). Males showed the highest ASRs with an increasing time trend until 2009 (APC = 6.9%, 95% CI: 1.5, 12.6, *p* = .017), remaining constant thereafter; an upward change until 2008, albeit non‐significant (*p* = .610) was observed in females (Figure 2).

The majority of breast cancer cases involved females with a significant (*p* < .05) APC steady increase in ASRs over the whole study period (APC = 1.2%, 95% CI: 0.5, 1.9, *p* = .002) (Figure 2). Males comprised a small fraction of the total new annual cancer cases, showing a downward change, albeit this trend was not significant (APC = −3.3%, 95% CI: −9.2, 3.0, *p* = .273).

For prostate cancer, non‐significant change in ASRs until 2012 was observed, followed by a significant (*p* < .05) reduction in the ASRs from 2012 onwards (APC = −3.4%, 95% CI: −6.4, −0.3, *p* = .035) (Figure 3). The overall AAPC was −0.8% (95% CI: −2.1, 0.6, *p* = .267).

Thyroid cancer ASRs showed a sharp increasing trend throughout the study period for the overall population (APC = 12.8%, 95% CI: 10.5, 15.2, *p* < .001) (Figure 3). The highest APC was observed among women (13.0%, 95% CI: 10.6, 15.5, *p* < .001), while similar trend was observed in males (APC = 11.6%, 95% CI: 8.2, 15.1, *p* < .001); the overall AAPC was 12.8% (95% CI: 10.5, 15.2, *p* < .001).

## 4 DISCUSSION

During the study period (2004–2017), increasing temporal trends were documented for thyroid and breast cancer age‐standardized incidence rates for both sexes and for females only, respectively, for the population of Cyprus. Lung cancer ASRs seemed to have stabilized (no increase or decrease) during the recent years (2009 onwards) for both sexes; a similar pattern was observed for colorectal cancer in males only. The ASRs of prostate cancer in men from 2012 onwards and the ASRs of colorectal cancer for females from 2007 onwards seemed to be in steady decline. The colorectal cancer ASR temporal patterns overall, during the whole study period were unchanged. This study builds upon the earlier comprehensive analysis of cancer incidence in Cyprus using 11 years of collected data (1998–2008).^23^, ^24^ Since 2008 and to the best of our knowledge, this is the only published time series analysis of ASRs for the five most frequent cancer sites of the general population of Cyprus (0–85+ years of age).

Thyroid cancer temporal trends in Cyprus are quite interesting, exhibiting a steep increase over the last 20 years or so, while also being among the highest ASR of thyroid cancer in the world (32.5 per 100 000 in Cyprus, 8 per 100 000 in EU^12^), affecting mostly females.^25^ In 2012, the European ASR for thyroid cancer was 6.3 cases per 100 000. Countries with the higher ASR were Lithuania (15.5), Italy (13.5), Austria (12.4), Croatia (11.4), and Luxembourg (11.1), while those with lower ASR were Montenegro (2.2), followed by Albania, Bosnia and Herzegovina and Greece (both 1.9).^25^ Out of 189 countries part of the GLOBOCAN database, Cyprus was among the top three countries, globally, with the highest incidence‐to‐mortality rate ratio for thyroid cancer in both sexes.^26^ A significant annual increase in the thyroid cancer incidence rates was documented for Cypriot children (beta = 0.12; 95% CI: 0.04, 0.21, *p* = .008) exhibiting a steady increase for children in Cyprus over the years (1998–2017).^27^ Thus, the increasing temporal patterns of thyroid cancer in adults reported in the present study (AAPC = 12.8, 95% CI: 10.5, 15.2, *p* < .001) were corroborated by the similarly increasing temporal trends for the childhood thyroid cancer (0–19 years of age) during the 1998–2017 study period.^26^

Based on the European cancer burden study, the 2020 estimate of breast cancer ASR was 140.4 per 100 000, being similar to the EU‐27 average (142.8).^5^ It is of note here that the incidence and mortality rates (2007–2016) for the below discussed cancer sites were projected to 2020 and applied to the 2020 population, including populations from both the Republic of Cyprus government‐controlled and non‐controlled areas.^4^ Higher breast cancer ASRs (per 100 000) were reported in Belgium (ASR, 194), the Netherlands (174.4), Luxemburg (171.6), Malta (171.3), and Denmark (171.2).^5^

Colorectal cancer was the second most frequently diagnosed cancer site in Europe,^5^ while in the present study it ranked third in Cyprus for both sexes (ASRs of 88.1 for males and 34 for females). The colorectal cancer ASR for males and females in Europe was 89 and 55, respectively, and the top three countries with the highest ASRs were Slovakia (141.3), Hungary (135.6) and Slovenia (133.3) for males, while for females, the top three countries were Norway (92.7), Denmark (83.9) and the Netherlands (78.9).^5^ This sexual dimorphism in the colorectal cancer ASRs was also noted in the present study.

Lung cancer ASR estimates for 2020 (102.1) in Cypriot males were similar to the EU average (97.6), while Cypriot females presented with a lower ASR (24.6) than the EU average (38.6).^4^ These estimates for Cyprus in 2020 were a bit higher than the lung cancer ASR calculated in the present study (~74 per 100 000), covering the period 2004–2017. For men, the highest ASR (per 100 000) were found in Hungary (138.3), Serbia (136.4), Bosnia and Herzegovina (131.3), Latvia (127.9), and Greece (127.2), while for females in Ireland (85.1), Denmark (85.1), Hungary (76.6), Iceland (74.3) and United Kingdom (71.4).^5^

Cyprus was among the countries with the highest prostate cancer ASR estimates for 2020 (199.6 per 100 000), including, Ireland (250.9), Estonia (245.4), Sweden (223.1), Norway (222.4), Latvia (219.2), and France (214.4).^5^ However, it appeared that prostate cancer ASR in Cyprus are in declining mode, since 2012, using the cancer registry data in the present study.

It is remarkable that higher ASR in females, almost double than that of males, was observed for all five most common cancer sites in Cyprus in the 20–64 years of age group, while the opposite was observed for the >65 years of age group, with males exhibiting double ASRs than for females. In the first age group (0–19 yrs), despite the low numbers of cancer incident cases and ASRs, girls exhibited higher ASRs than boys.

The etiology of carcinogenesis for these five most frequent cancer sites in Cyprus is at large unclear. Globally, in 2019, the leading risk factors for risk‐attributable cancer deaths and cancer incidence (disability adjusted life years, DALYs) for both sexes combined were smoking, followed by alcohol use, and high body mass index (BMI).^28^ Historic evidence of smoking in Cyprus suggests a decreasing pattern over the recent years. Back in 1989, smoking prevalence in men was 43%, while the smoking prevalence in the 2003 European Health Survey (EHS) in Cyprus survey was 38% for men; for women, the corresponding smoking prevalence was 10.5%.^23^ During the 2008 survey in Cyprus, it was shown that nearly 33% of the Cyprus urban population reported smoking.^29^ In 2014, based on the same Cyprus survey, there were 38% men and 14.1% female daily smokers,^30^ while the same survey conducted in 2019 showed a slight decrease in males with 30% daily smokers and 12.8% in females.^31^ Epidemiological studies showed higher rates of tobacco‐related cancers in males than females,^29^ corroborating the sex distribution in smoking status of the Cypriot population in this study and elsewhere.

Thyroid cancer temporal trends during the last 3–4 decades in Cyprus are remarkable, suggesting that environmental (non‐genetic) risk factors may be implicated with the disease process. Breast cancer is the most common cancer in Cyprus with the highest ASR, exhibiting also a steady rising temporal trend (2004–2017) that was earlier (2000–2008) documented for Cyprus.^32^ These temporal patterns for thyroid and breast cancer sites would be only partially explained by the increasing use of needle biopsies^33^ or the establishment of free mammography screening programs that began in Cyprus back in 2003 and expanded throughout the island by 2007 for women aged 50–65.^33^ An early age at menarche can also be a risk factor as breast starts mitotic activity early which would increase breast cancer risk.^34^, ^35^

Some environmental risk factors which may be possible contributors to the documented increase in some hormonal cancers, such as breast and thyroid cancer, including, high BMI, environmental pollutants, such as endocrine disruptors, unhealthy diets and ionizing radiation.^15^ Worth mentioning is the potential influence of environmental factors (unhealthy lifestyle/behaviors, unhealthy eating, high body mass index, endocrine‐disrupting chemicals, etc.) in carcinogenesis that would potentially contribute to thyroid cancer disease process, especially during critical windows of susceptibility, such as in pregnancy, and during early life period for infants and toddlers. Further, obesity has been charged with the development of certain hormonal cancer types,^28^ affecting a large percentage of the Cypriot population; In 2019, the obesity and overweight percentages in Cyprus were 41.9% and 26.3% for men and women respectively,^31^ while Cyprus has been historically placed in the top #3 of the European countries with the highest childhood obesity rates.^36^

There was a temporal decrease in colorectal cancer ASRs over the years in this study. In effect, this would be partially attributed to secondary prevention programs already in place for the Cypriot population. According to the Nicosia General Hospital, colonoscopies implemented in 2011 showed a 62% increase when compared with those in 2007.^37^

A decrease over time was also reported for prostate cancer ASRs, but this trend is not straightforward to explain. The PSA test (Prostate Specific Antigen test) is widely used for prostate cancer screening purposes, but it was not officially used as a population screening tool during the study period in Cyprus. However, the Cyprus Ministry of Health intends soon to apply the PSA test for men, 50–60 years of age.^38^

This study used official cancer registry data to study the time trends of the most frequently occurring cancer types in Cyprus, covering the whole general population for a 13‐year period. A limitation was that an additional time series analysis by every geographical district separately was not feasible, due to the small Cyprus population. The joinpoint modeling may imply that cancer rates change at a constant rate for a fixed period, and then suddenly change direction, but this is not necessarily accurate, but rather a simplified realization. The observed temporal declines in certain cancer sites were not related to lags in reporting of cancer cases; full data was reported for the whole study period on time. Considering the nature of the CyCR registration process, it appears that data on cancer cases may not be published, if the registration year hasn't been completed.

However, little if any information is available for the spatiotemporal patterns of biologically plausible risk factors (e.g., metabolic, behavioral or environmental) of carcinogenesis in Cyprus, and their association with the disease process of thyroid and breast cancer. It is warranted that more emphasis on the characteristics and distribution of hormonal cancer risk determinants in the population at risk, such as lifestyle and behavioral factors shall be placed, if we were to better understand the environmental origins of thyroid and breast carcinogenesis. This study's dataset would feed into the evaluation and fine tuning of ongoing cancer surveillance and control strategic programs that focus on both prevention, early detection and treatment. Such knowledge may be particularly useful for cancer sites of higher mortality or for those with persistently upwards temporal trends.

## 5 CONCLUSION

During the 2004–2017 study period, thyroid and breast cancer ASRs demonstrated a rising temporal trend, but this was not the case for prostate, colorectal and lung cancer as they appeared to either stabilize or decline with time for the general population of Cyprus (0–85+ years of age). Secondary prevention programs and improved access to healthcare might have contributed to the above‐mentioned downward trends of colorectal cancer in Cyprus. The percentage of active smokers in Cyprus is also in decline over the recent years explaining part of the temporally stabilized lung cancer ASRs. Such knowledge coming from national cancer registry databases is an important basis for cancer prevention, passive/active surveillance schemes and control strategies for the general population of the Republic of Cyprus.

## AUTHOR CONTRIBUTIONS

**Anastasia Spartiati:** Formal analysis (lead); investigation (equal); visualization (equal); writing – original draft (equal); writing – review and editing (equal). **Anna Demetriou:** Data curation (equal); project administration (equal); resources (equal); writing – original draft (equal); writing – review and editing (equal). **V. Scoutellas:** Investigation (equal); project administration (equal); resources (equal); supervision (equal); writing – original draft (equal); writing – review and editing (equal). **Costas A. Christophi:** Conceptualization (equal); data curation (equal); investigation (equal); project administration (equal); resources (equal); software (equal); supervision (equal); writing – original draft (equal); writing – review and editing (equal). **Konstantinos C. Makris:** Conceptualization (equal); data curation (equal); funding acquisition (equal); investigation (equal); methodology (equal); project administration (equal); resources (equal); supervision (equal); validation (equal); writing – original draft (equal); writing – review and editing (equal).

## CONFLICT OF INTEREST STATEMENT

The authors have stated explicitly that there are no conflicts of interest in connection with this article.

## ETHICS STATEMENT

Approval of the research protocol by an Institutional Reviewer Board: Cyprus National Bioethics Committee (EEBΚ/ΕΠ/2019/01/171).

## Supporting information


**Table S1.** Cancer epidemiology by stage, histology, and smoking status for all cancer sites in Cyprus between 2004 and 2017, overall and stratified by sex.
**Table S2.** Annual Percentage Change (APC) and Average Annual Percentage Change (AAPC) of the age standardized rates (ASR) for the five most common cancer sites in Cyprus, for both sexes, and overall.

## Data Availability

The data that support the findings of this study are available upon reasonable request by the Cyprus Ministry of Health, Cancer Registry. Further information and requests may be directed to the corresponding author.
