# Supplementary material for: Temporal trends of cancer incidence rates for the most frequent cancer sites in Cyprus (2004–2017)
Source: Cancer Rep (Hoboken). 2024 Jun 12;7(6):e2000. doi: 10.1002/cnr2.2000 (PMC11168067; doi:10.1002/cnr2.2000)
Supplement: Supplementary file 1 — Table S1. Cancer epidemiology by stage, histology, and smoking status for all cancer sites in Cyprus between 2004 and 2017, overall and stratified by sex. Table S2. Annual Percentage Change (APC) and Average Annual Percentage Change (AAPC) of the age standardized rates (ASR) for the five most common cancer sites in Cyprus, for both sexes, and overall. [file CNR2-7-e2000-s001.docx]

**SUPPLEMENTARY INFORMATION**

**Temporal trends of cancer incidence rates for the most frequent cancer sites in Cyprus (2004-2017)**

*Anastasia Spartiati^1^, Anna Demetriou^2^, V. Scoutellas^2^, Costas A. Christophi^1^ and Konstantinos C. Makris^1^**

^1^ Cyprus International Institute for Environmental and Public Health, School of Health Sciences, Cyprus University of Technology, Limassol, Cyprus

^2^ Cancer Registry, Ministry of Health, Nicosia, Cyprus

|  | **Table S1.** Cancer epidemiology by stage, histology, and smoking status for all cancer sites in Cyprus between 2004-2017, overall and stratified by sex. | | | | | | | | | | |  |  |  |
| --- | --- | --- | --- | --- | --- | --- | --- | --- | --- | --- | --- | --- | --- | --- |
|  | | **Overall** | | | | | **Males** | | | | **Females** | | | |
|  | | **N (%)** | **cIR** | | | **ASR** | **N (%)** | **cIR** | | **ASR** | **N (%)** | **cIR** | | **ASR** |
| **Cancer Stage** | | | | | | | | | | | | | | |
| 0= In Situ (intraepithelial non-invasive) | | 2 (0.007) | | 0 | 0 | | 1 (0.01) | 0 | 0 | | 1 (0.01) | 0 | 0 | |
| 1= Local only (Stage I for lymphomas) | | 9522  (35.24) | | 1133 | 1385 | | 3900 (28.97) | 954 | 1313 | | 5621 (42.03) | 1303 | 463 | |
| 2= Regional with direct extension only | | 2602  (9.6) | | 310 | 398 | | 1561 (11.59) | 382 | 517 | | 1041 (7.78) | 241 | 291 | |
| 3= Regionally affected lymph nodes only | | 2665 (9.93) | | 317 | 366 | | 320 (2.38) | 78 | 98 | | 2345 (17.54) | 543 | 612 | |
| 4= Regional with direct and through the lymph nodes extension | | 1976 (7.31) | | 235 | 296 | | 913 (6.78) | 223 | 299 | | 1063 (7.95) | 246 | 292 | |
| 5= Regional not otherwise specified (Stage II for lymphomas) | | 3 (0.01) | | 0 | 1 | | 2 (0.01) | 0 | 1 | | 1 (0.01) | 0 | 0 | |
| 7= Distant (Stage III or IV for lymphomas) | | 4453 (16.48) | | 530 | 686 | | 2971 (22.07) | 727 | 988 | | 1482 (11.08) | 343 | 422 | |
| 9= Unknown, unspecified | | 5794 (21.44) | | 690 | 991 | | 3795 (28.19) | 972 | 1533 | | 1819 (13.6) | 421 | 548 | |
| **Smoking** | | | | | | | | | | | | | | |
| Current | | 4241  (15.7) | | 505 | 611 | | 2845 (20.85) | 696 | 903 | | 1396 (10.44) | 323 | 347 | |
| Former | | 3205 (11.86) | | 381 | 513 | | 2839 (20.81) | 695 | 983 | | 366 (2.74) | 85 | 98 | |
| Never | | 7555 (27.96) | | 899 | 1082 | | 2250 (16.49) | 550 | 745 | | 5305 (39.66) | 1229 | 1398 | |
| Uknown | | 12016 (44.48) | | 1430 | 1917 | | 5709 (41.85) | 1397 | 2120 | | 6307 (47.16) | 1461 | 1788 | |
| **Histology** | | | | | | | | | | | | | | |
| Neoplasm | | 3096 (11.46) | | 368 | 525 | | 1895 (13.9) | 464 | 725 | | 1201 (8.98) | 278 | 368 | |
| Carcinoma | | 23879 (88.38) | | 2842 | 3592 | | 11734 (86) | 2871 | 4020 | | 12145 (90.82) | 2814 | 3255 | |
| Sarcoma | | 19 (0.07) | | 2 | 2 | | 11 (0.08) | 3 | 4 | | 8 (0.06) | 2 | 2 | |
| Others | | 23 (0.09) | | 3 | 3 | | 3 (0.02) | 1 | 1 | | 20 (0.14) | 5 | 5 | |

|  |
| --- |
| \|  \| \|  \|  \|  \|  \| **Table S2.** Annual Percentage Change (APC) and Average Annual Percentage Change (AAPC) of the cancer Age Standardize Rate (ASR) for the five most common cancer sites in Cyprus, for both sexes (prostate cancer excluded) and overall population. \|  \|  \| \| --- \| --- \| --- \| --- \| --- \| --- \| --- \| \|  \| \| **Estimated Joinpoints** \| \| \| \| \| \| --- \| --- \| --- \| --- \| --- \| \| **Cohort** \| **Joinpoint** \| **Estimate** \| **Lower CI** \| **Upper CI** \| \| C18-C20 Females \| 1 \| 2007 \| 2006 \| 2012 \| \| C18-C20 Males \| 1 \| 2012 \| 2006 \| 2015 \| \| C18-C20 Total \| 1 \| 2010 \| 2006 \| 2015 \| \| C34 Females \| 1 \| 2008 \| 2006 \| 2015 \| \| C34 Males \| 1 \| 2009 \| 2007 \| 2014 \| \| C34 Total \| 1 \| 2009 \| 2007 \| 2013 \| \| C61 Males \| 1 \| 2012 \| 2009 \| 2014 \| \| \| \| \|  \|  \| \|  \|  \|  \|  \|  \|  \|  \| \|  \| \| **Annual Percent Change (APC)** \| \| \| \| \| \| \| \| \| \| --- \| --- \| --- \| --- \| --- \| --- \| --- \| --- \| --- \| \| **Cohort** \| **Segment** \| **Lower EndPoint** \| **Upper Endpoint** \| **APC** \| **Lower CI** \| **Upper CI** \| **Test Statistic (t)** \| **Prob > \|t\|** \| \| C18-C20 Females \| 1 \| 2004 \| 2007 \| 7,7 \| -4,0 \| 20,8 \| 1,5 \| 0,178 \| \| C18-C20 Females \| 2 \| 2007 \| 2017 \| -3,0* \| -4,6 \| -1,4 \| -4,2 \| 0,002 \| \| C18-C20 Males \| 1 \| 2004 \| 2012 \| 2,7* \| 0,1 \| 5,4 \| 2,3 \| 0,046 \| \| C18-C20 Males \| 2 \| 2012 \| 2017 \| -3,9 \| -8,6 \| 0,9 \| -1,9 \| 0,097 \| \| C18-C20 Total \| 1 \| 2004 \| 2010 \| 2,5 \| -1,5 \| 6,7 \| 1,4 \| 0,195 \| \| C18-C20 Total \| 2 \| 2010 \| 2017 \| -2,7 \| -5,4 \| 0,1 \| -2,1 \| 0,060 \| \| C34 Females \| 1 \| 2004 \| 2008 \| 6,0 \| -5,9 \| 19,3 \| 1,1 \| 0,300 \| \| C34 Females \| 2 \| 2008 \| 2017 \| 0,7 \| -2,1 \| 3,5 \| 0,5 \| 0,610 \| \| C34 Males \| 1 \| 2004 \| 2009 \| 6,9* \| 1,5 \| 12,6 \| 2,9 \| 0,017 \| \| C34 Males \| 2 \| 2009 \| 2017 \| -0,1 \| -2,1 \| 2,0 \| -0,1 \| 0,920 \| \| C34 Total \| 1 \| 2004 \| 2009 \| 6,6* \| 1,6 \| 11,8 \| 3,0 \| 0,014 \| \| C34 Total \| 2 \| 2009 \| 2017 \| 0,3 \| -1,7 \| 2,2 \| 0,3 \| 0,775 \| \| C50 Females \| 1 \| 2004 \| 2017 \| 1,2* \| 0,5 \| 1,9 \| 4,0 \| 0,002 \| \| C50 Males \| 1 \| 2004 \| 2017 \| -3,3 \| -9,2 \| 3,0 \| -1,1 \| 0,273 \| \| C50 Total \| 1 \| 2004 \| 2017 \| 1,2* \| 0,5 \| 1,8 \| 3,8 \| 0,003 \| \| C61 Males \| 1 \| 2004 \| 2012 \| 0,9 \| -0,8 \| 2,6 \| 1,2 \| 0,266 \| \| C61 Males \| 2 \| 2012 \| 2017 \| -3,4* \| -6,4 \| -0,3 \| -2,5 \| 0,035 \| \| C73 Females \| 1 \| 2004 \| 2017 \| 13,0* \| 10,6 \| 15,5 \| 12,3 \| < 0,001 \| \| C73 Males \| 1 \| 2004 \| 2017 \| 11,6* \| 8,2 \| 15,1 \| 7,8 \| < 0,001 \| \| C73 Total \| 1 \| 2004 \| 2017 \| 12,8* \| 10,5 \| 15,2 \| 12,6 \| < 0,001 \| \| \| \| \| \|  \| \|  \|  \| \| * Indicates that the Annual Percent Change (APC) is significantly different from zero at the alpha = 0.05 level \| \| --- \| \| \| \| \|  \| \|  \|  \|  \|  \| \| - The statistic could not be calculated. \| \| --- \| \| \|  \| \|  \|  \|  \|  \|  \|  \|  \| \|  \| \| **Average Annual Percent Change (AAPC)** \| \| \| \| \| \| \| \| \| \| --- \| --- \| --- \| --- \| --- \| --- \| --- \| --- \| --- \| \| **Cohort** \| **Range** \| **Lower EndPoint** \| **Upper Endpoint** \| **AAPC** \| **Lower CI** \| **Upper CI** \| **Test Statistic *** \| **P-Value *** \| \| C18-C20 Females \| Full Range \| 2004 \| 2017 \| -0,7 \| -3,2 \| 1,9 \| -0,5 \| 0,612 \| \| C18-C20 Males \| Full Range \| 2004 \| 2017 \| 0,1 \| -2,0 \| 2,3 \| 0,1 \| 0,931 \| \| C18-C20 Total \| Full Range \| 2004 \| 2017 \| -0,3 \| -2,4 \| 1,8 \| -0,3 \| 0,773 \| \| C34 Females \| Full Range \| 2004 \| 2017 \| 2,3 \| -1,3 \| 6,0 \| 1,2 \| 0,223 \| \| C34Males \| Full Range \| 2004 \| 2017 \| 2,5* \| 0,5 \| 4,7 \| 2,4 \| 0,016 \| \| C34 Total \| Full Range \| 2004 \| 2017 \| 2,6* \| 0,7 \| 4,6 \| 2,7 \| 0,007 \| \| C50 Females \| Full Range \| 2004 \| 2017 \| 1,2* \| 0,5 \| 1,9 \| 4,0 \| 0,002 \| \| C50 Males \| Full Range \| 2004 \| 2017 \| -3,3 \| -9,2 \| 3,0 \| -1,1 \| 0,273 \| \| C50 Total \| Full Range \| 2004 \| 2017 \| 1,2* \| 0,5 \| 1,8 \| 3,8 \| 0,003 \| \| C61 Males \| Full Range \| 2004 \| 2017 \| -0,8 \| -2,1 \| 0,6 \| -1,1 \| 0,267 \| \| C73 Females \| Full Range \| 2004 \| 2017 \| 13,0* \| 10,6 \| 15,5 \| 12,3 \| < 0,001 \| \| C73 Males \| Full Range \| 2004 \| 2017 \| 11,6* \| 8,2 \| 15,1 \| 7,8 \| < 0,001 \| \| C73 Total \| Full Range \| 2004 \| 2017 \| 12,8* \| 10,5 \| 15,2 \| 12,6 \| < 0,001 \| \| \| \| \| \|  \| \|  \|  \|  \|  \|  \|  \|  \| \|  \|  \|  \| \| * Indicates that the AAPC is significantly different from zero at the alpha = 0.05 level. ~ If the AAPC is within one segment, the t-distribution is used. Otherwise, the normal (z) distribution is used.  - The statistic could not be calculated. \| \| --- \| \| \| \|  \| \| \|  \|  \| \| --- \| --- \| --- \| --- \| --- \| --- \| --- \| --- \| --- \| --- \| --- \| --- \| --- \| --- \| --- \| --- \| --- \| --- \| --- \| --- \| --- \| --- \| --- \| --- \| --- \| --- \| --- \| --- \| --- \| --- \| --- \| --- \| --- \| --- \| --- \| --- \| --- \| --- \| --- \| --- \| --- \| --- \| --- \| --- \| --- \| --- \| --- \| --- \| --- \| --- \| --- \| --- \| --- \| --- \| --- \| --- \| --- \| --- \| --- \| --- \| --- \| --- \| --- \| --- \| --- \| --- \| --- \| --- \| --- \| --- \| --- \| --- \| --- \| --- \| --- \| --- \| --- \| --- \| --- \| --- \| --- \| --- \| --- \| --- \| --- \| --- \| --- \| --- \| --- \| --- \| --- \| --- \| --- \| --- \| --- \| --- \| --- \| --- \| --- \| --- \| --- \| --- \| --- \| --- \| --- \| --- \| --- \| --- \| --- \| --- \| --- \| --- \| --- \| --- \| --- \| --- \| --- \| --- \| --- \| --- \| --- \| --- \| --- \| --- \| --- \| --- \| --- \| --- \| --- \| --- \| --- \| --- \| --- \| --- \| --- \| --- \| --- \| --- \| --- \| --- \| --- \| --- \| --- \| --- \| --- \| --- \| --- \| --- \| --- \| --- \| --- \| --- \| --- \| --- \| --- \| --- \| --- \| --- \| --- \| --- \| --- \| --- \| --- \| --- \| --- \| --- \| --- \| --- \| --- \| --- \| --- \| --- \| --- \| --- \| --- \| --- \| --- \| --- \| --- \| --- \| --- \| --- \| --- \| --- \| --- \| --- \| --- \| --- \| --- \| --- \| --- \| --- \| --- \| --- \| --- \| --- \| --- \| --- \| --- \| --- \| --- \| --- \| --- \| --- \| --- \| --- \| --- \| --- \| --- \| --- \| --- \| --- \| --- \| --- \| --- \| --- \| --- \| --- \| --- \| --- \| --- \| --- \| --- \| --- \| --- \| --- \| --- \| --- \| --- \| --- \| --- \| --- \| --- \| --- \| --- \| --- \| --- \| --- \| --- \| --- \| --- \| --- \| --- \| --- \| --- \| --- \| --- \| --- \| --- \| --- \| --- \| --- \| --- \| --- \| --- \| --- \| --- \| --- \| --- \| --- \| --- \| --- \| --- \| --- \| --- \| --- \| --- \| --- \| --- \| --- \| --- \| --- \| --- \| --- \| --- \| --- \| --- \| --- \| --- \| --- \| --- \| --- \| --- \| --- \| --- \| --- \| --- \| --- \| --- \| --- \| --- \| --- \| --- \| --- \| --- \| --- \| --- \| --- \| --- \| --- \| --- \| --- \| --- \| --- \| --- \| --- \| --- \| --- \| --- \| --- \| --- \| --- \| --- \| --- \| --- \| --- \| --- \| --- \| --- \| --- \| --- \| --- \| --- \| --- \| --- \| --- \| --- \| --- \| --- \| --- \| --- \| --- \| --- \| --- \| --- \| --- \| --- \| --- \| --- \| --- \| --- \| --- \| --- \| --- \| --- \| --- \| --- \| --- \| --- \| --- \| --- \| --- \| --- \| --- \| --- \| --- \| --- \| --- \| --- \| --- \| --- \| --- \| --- \| --- \| --- \| --- \| --- \| --- \| --- \| --- \| --- \| --- \| --- \| --- \| --- \| --- \| --- \| --- \| --- \| --- \| --- \| --- \| --- \| --- \| --- \| --- \| --- \| --- \| --- \| --- \| --- \| --- \| --- \| --- \| --- \| --- \| --- \| --- \| --- \| --- \| --- \| --- \| --- \| --- \| --- \| --- \| --- \| --- \| --- \| --- \| --- \| --- \| --- \| --- \| --- \| --- \| --- \| --- \| --- \| --- \| --- \| --- \| --- \| --- \| --- \| --- \| --- \| --- \| --- \| --- \| --- \| --- \| --- \| --- \| --- \| --- \| --- \| --- \| --- \| --- \| --- \| --- \| --- \| --- \| --- \| --- \| --- \| --- \| --- \| --- \| --- \| --- \| --- \| --- \| --- \| --- \| \|  \|  \|  \|  \|  \| |

**CANCER SITE JOINPOINT MODELS**

**
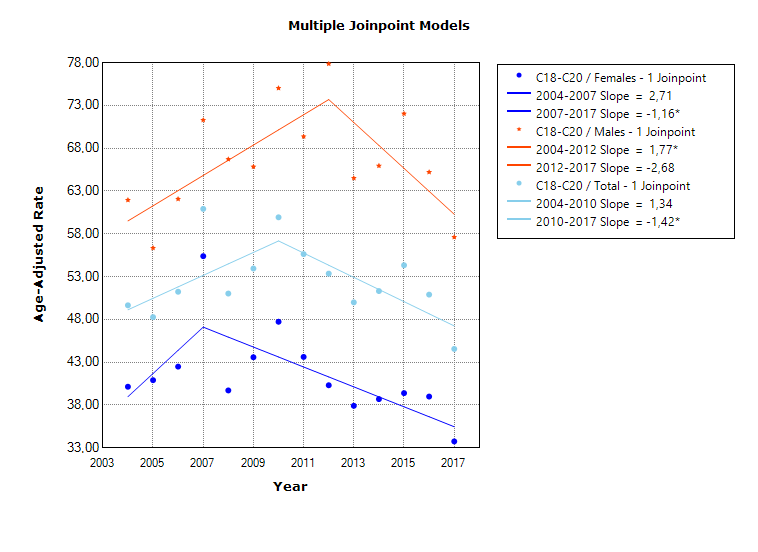
Colorectal Cancer**

| **Model Statistics for Colorectal Cancer** | | | | | | | | | | | | | | | | |  |
| --- | --- | --- | --- | --- | --- | --- | --- | --- | --- | --- | --- | --- | --- | --- | --- | --- | --- |
| **Cohort** | **Number of Joinpoints** | | **Number of Observations** | **Number of Parameters** | | | **Degrees of Freedom** | **Sum of Squared Errors** | | | **Mean Squared Error** | | | **Autocorrelation**  **Parameter** | | |  |
| C18-C20 females | 1 | | 14 | 4 | | | 10 | 10,93 | | | 1,10 | | | Uncorrelated | | |  |
| C18-C20 males | 1 | | 14 | 4 | | | 10 | 9,70 | | | 0,97 | | | Uncorrelated | | |  |
| C18-C20 total | 1 | | 14 | 4 | | | 10 | 14,80 | | | 1,48 | | | Uncorrelated | | |  |
| **Estimated Joinpoints** | | | | | | | | | | | | | | | |  |  |
| **Cohort** | | **Joinpoint** | | | **Estimate** | | | | | **Lower CI** | | | **Upper CI** | | |  |  |
| C18-C20 females | | 1 | | | 2007 | | | | | 2006 | | | 2015 | | |  |  |
| C18-C20 males | | 1 | | | 2012 | | | | | 2006 | | | 2015 | | |  |  |
| C18-C20 total | | 1 | | | 2010 | | | | | 2006 | | | 2015 | | |  |  |
| **Estimated Regression Coefficients (Beta)** | | | | | | | | | | | | | | | | | |
| **Standard Parameterization** | | | | | | | | | | | | | | | | | |
| **Cohort** | | **Parameter** | | | | **Param Estimate** | | | **Standard Error** | | | **Test Statistic (t)** | | | **Prob > \|t\|** | | |
| C18-C20 females | | Intercept 1 | | | | -5390,22 | | | 4156,08 | | | -1,30 | | | 0,23 | | |
| C18-C20 females | | Slope 1 | | | | 2,71 | | | 2,07 | | | 1,31 | | | 0,22 | | |
| C18-C20 females | | Slope 2 - Slope 1 | | | | -3,87 | | | 2,09 | | | -1,85 | | | 0,10 | | |
| C18-C20 males | | Intercept 1 | | | | -3493,84 | | | 1478,52 | | | -2,36 | | | 0,04 | | |
| C18-C20 males | | Slope 1 | | | | 1,77 | | | 0,74 | | | 2,41 | | | 0,04 | | |
| C18-C20 males | | Slope 2 - Slope 1 | | | | -4,45 | | | 1,51 | | | -2,95 | | | 0,02 | | |
| C18-C20 total | | Intercept 1 | | | | -2637,40 | | | 1768,09 | | | -1,49 | | | 0,17 | | |
| C18-C20 total | | Slope 1 | | | | 1,34 | | | 0,88 | | | 1,52 | | | 0,16 | | |
| C18-C20 total | | Slope 2 - Slope 1 | | | | -2,76 | | | 1,07 | | | -2,57 | | | 0,03 | | |
| **General Parameterization** | | | | | | | | | | | | | | | | | |
| **Cohort** | | **Parameter** | | | | **Param Estimate** | | | **Standard Error** | | | **Test Statistic (t)** | | | **Prob > \|t\|** | | |
| C18-C20 females | | Intercept 1 | | | | -5390,22 | | | 4156,08 | | | -1,30 | | | 0,23 | | |
| C18-C20 females | | Intercept 2 | | | | 2382,48 | | | 576,47 | | | 4,13 | | | 0,00 | | |
| C18-C20 females | | Slope 1 | | | | 2,71 | | | 2,07 | | | 1,31 | | | 0,22 | | |
| C18-C20 females | | Slope 2 | | | | -1,16* | | | 0,29 | | | -4,06 | | | 0,00 | | |
| C18-C20 males | | Intercept 1 | | | | -3493,84 | | | 1478,52 | | | -2,36 | | | 0,04 | | |
| C18-C20 males | | Intercept 2 | | | | 5468,02 | | | 2659,95 | | | 2,06 | | | 0,07 | | |
| C18-C20 males | | Slope 1 | | | | 1,77* | | | 0,74 | | | 2,41 | | | 0,04 | | |
| C18-C20 males | | Slope 2 | | | | -2,68 | | | 1,32 | | | -2,03 | | | 0,07 | | |
| C18-C20 total | | Intercept 1 | | | | -2637,40 | | | 1768,09 | | | -1,49 | | | 0,17 | | |
| C18-C20 total | | Intercept 2 | | | | 2907,71 | | | 1238,22 | | | 2,35 | | | 0,04 | | |
| C18-C20 total | | Slope 1 | | | | 1,34 | | | 0,88 | | | 1,52 | | | 0,16 | | |
| C18-C20 total | | Slope 2 | | | | -1,42* | | | 0,61 | | | -2,31 | | | 0,05 | | |


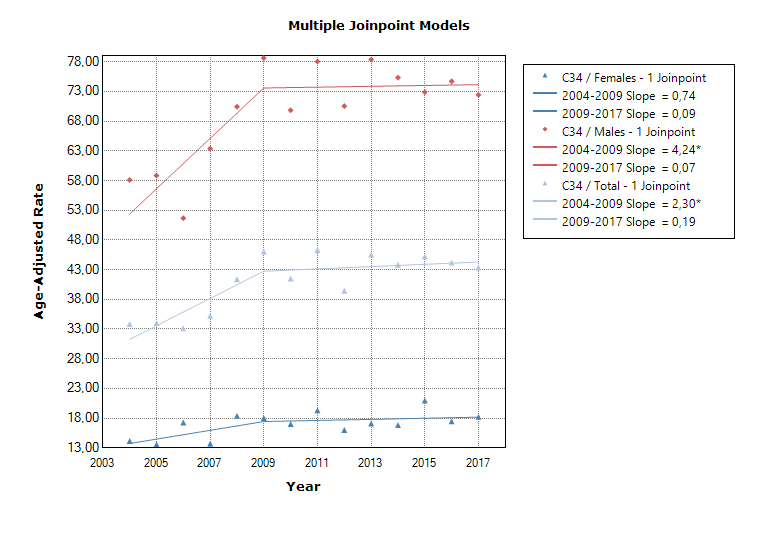
**Lung Cancer**

| **Model Statistics for Lung Cancer** | | | | | | | | | | | | | | | | |  |
| --- | --- | --- | --- | --- | --- | --- | --- | --- | --- | --- | --- | --- | --- | --- | --- | --- | --- |
| **Cohort** | **Number of Joinpoints** | **Number of Observations** | | **Number of Parameters** | | | **Degrees of Freedom** | | **Sum of Squared Errors** | | **Mean Squared Error** | | | **Autocorrelation**  **Parameter** | | |  |
| C34 females | 1 | 14 | | 4 | | | 10 | | 6,34 | | 0,63 | | | Uncorrelated | | |  |
| C34 males | 1 | 14 | | 4 | | | 10 | | 9,97 | | 1,00 | | | Uncorrelated | | |  |
| C34 total | 1 | 14 | | 4 | | | 10 | | 11,28 | | 1,13 | | | Uncorrelated | | |  |
| **Estimated Joinpoints** | | | | | | | | | | | | | | | | | |
| **Cohort** | | | | | **Joinpoint** | | | **Estimate** | | | | **Lower CI** | | | | **Upper CI** | |
| C34 females | | | | | 1 | | | 2009 | | | | 2006 | | | | 2015 | |
| C34 males | | | | | 1 | | | 2009 | | | | 2007 | | | | 2015 | |
| C34 total | | | | | 1 | | | 2009 | | | | 2007 | | | | 2015 | |
| **Estimated Regression Coefficients (Beta)** | | | | | | | | | | | | | | | | | |
| **Standard Parameterization** | | | | | | | | | | | | | | | | | |
| **Cohort** | | | **Parameter** | | | **Param Estimate** | | | | **Standard Error** | | | **Test Statistic (t)** | | **Prob > \|t\|** | | |
| C34 females | | | Intercept 1 | | | -1466,30 | | | | 1151,74 | | | -1,27 | | 0,23 | | |
| C34 females | | | Slope 1 | | | 0,74 | | | | 0,57 | | | 1,29 | | 0,23 | | |
| C34 females | | | Slope 2 - Slope 1 | | | -0,65 | | | | 0,64 | | | -1,02 | | 0,34 | | |
| C34 males | | | Intercept 1 | | | -8448,87 | | | | 3025,01 | | | -2,79 | | 0,02 | | |
| C34 males | | | Slope 1 | | | 4,24 | | | | 1,51 | | | 2,81 | | 0,020 | | |
| C34 males | | | Slope 2 - Slope 1 | | | -4,17 | | | | 1,66 | | | -2,51 | | 0,03 | | |
| C34 total | | | Intercept 1 | | | -4570,38 | | | | 1578,11 | | | -2,90 | | 0,018 | | |
| C34 total | | | Slope 1 | | | 2,30 | | | | 0,79 | | | 2,92 | | 0,02 | | |
| C34 total | | | Slope 2 - Slope 1 | | | -2,10 | | | | 0,87 | | | -2,42 | | 0,04 | | |
| **General Parameterization** | | | | | | | | | | | | | | | | | |
| **Cohort** | | | **Parameter** | | | **Param Estimate** | | | | **Standard Error** | | | **Test Statistic (t)** | | **Prob > \|t\|** | | |
| C34 females | | | Intercept 1 | | | -1466,30 | | | | 1151,74 | | | -1,27 | | 0,23 | | |
| C34 females | | | Intercept 2 | | | -168,71 | | | | 551,86 | | | -0,31 | | 0,77 | | |
| C34 females | | | Slope 1 | | | 0,74 | | | | 0,57 | | | 1,29 | | 0,23 | | |
| C34 females | | | Slope 2 | | | 0,09 | | | | 0,27 | | | 0,34 | | 0,74 | | |
| C34 males | | | Intercept 1 | | | -8448,87 | | | | 3025,01 | | | -2,79 | | 0,02 | | |
| C34 males | | | Intercept 2 | | | -68,88 | | | | 1398,51 | | | -0,05 | | 0,96 | | |
| C34 males | | | Slope 1 | | | 4,24* | | | | 1,51 | | | 2,81 | | 0,02 | | |
| C34 males | | | Slope 2 | | | 0,07 | | | | 0,69 | | | 0,10 | | 0,92 | | |
| C34 total | | | Intercept 1 | | | -4570,38 | | | | 1578,11 | | | -2,90 | | 0,02 | | |
| C34 total | | | Intercept 2 | | | -343,45 | | | | 752,51 | | | -0,46 | | 0,66 | | |
| C34 total | | | Slope 1 | | | 2,30* | | | | 0,79 | | | 2,92 | | 0,02 | | |
| C34 total | | | Slope 2 | | | 0,19 | | | | 0,37 | | | 0,51 | | 0,62 | | |

**
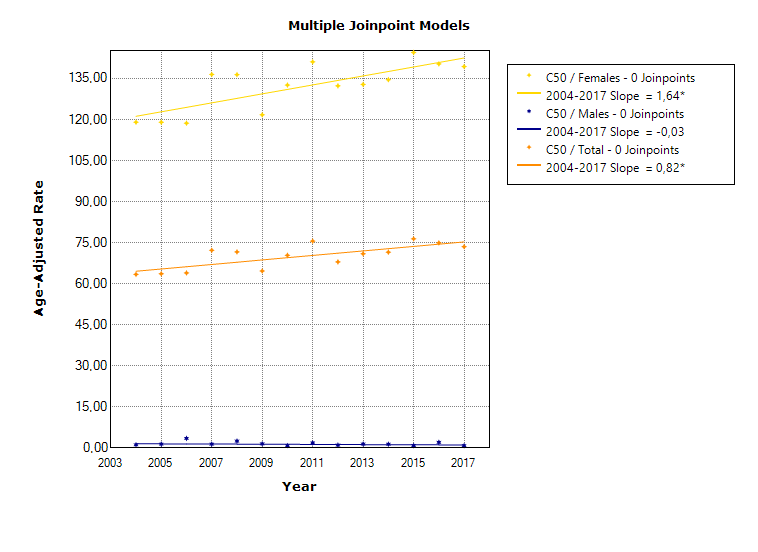
Breast Cancer**

| **Model Statistics for Breast Cancer** | | | | | | | | | | | | | | | |  |  |  |
| --- | --- | --- | --- | --- | --- | --- | --- | --- | --- | --- | --- | --- | --- | --- | --- | --- | --- | --- |
| **Cohort** | **Number of Joinpoints** | **Number of Observations** | | | **Number of Parameters** | | **Degrees of Freedom** | **Sum of Squared Errors** | | | **Mean Squared Error** | | **Autocorrelation**  **Parameter** | | |  |  |  |
| C50 females | 0 | 14 | | | 2 | | 12 | 12,16 | | | 1,01 | | Uncorrelated | | |  |  |  |
| C50 males | 0 | 14 | | | 2 | | 12 | 8,60 | | | 0,72 | | Uncorrelated | | |  |  |  |
| C50 total | 0 | 14 | | | 2 | | 12 | 12,10 | | | 1,01 | | Uncorrelated | | |  |  |  |
| **Estimated Regression Coefficients (Beta)** | | | | | | | | | | | | | | | | | | |
| **Standard Parameterization** | | | | | | | | | | | | | | | | | | |
| **Cohort** | | | | **Parameter** | | | | | **Param Estimate** | | | | | **Standard Error** | | **Test Statistic (t)** | **Prob > \|t\|** | |
| C50 females | | | | Intercept 1 | | | | | -3164,00 | | | | | 779,23 | | -4,06 | 0,00 | |
| C50 females | | | | Slope 1 | | | | | 1,64 | | | | | 0,39 | | 4,23 | 0,00 | |
| C50 males | | | | Intercept 1 | | | | | 67,91 | | | | | 68,98 | | 0,98 | 0,34 | |
| C50 males | | | | Slope 1 | | | | | -0,033 | | | | | 0,03 | | -0,97 | 0,35 | |
| C50 total | | | | Intercept 1 | | | | | -1586,51 | | | | | 413,85 | | -3,83 | 0,00 | |
| C50 total | | | | Slope 1 | | | | | 0,82 | | | | | 0,21 | | 4,00 | 0,00 | |
| **General Parameterization** | | | | | | | | | | | | | | | | | |  |
| **Cohort** | | | **Parameter** | | | **Param Estimate** | | | | **Standard Error** | | **Test Statistic (t)** | | | **Prob > \|t\|** | | |  |
| C50 females | | | Intercept 1 | | | -3164,00 | | | | 779,23 | | -4,06 | | | 0,00 | | |  |
| C50 females | | | Slope 1 | | | 1,64* | | | | 0,39 | | 4,23 | | | 0,00 | | |  |
| C50 males | | | Intercept 1 | | | 67,91 | | | | 68,98 | | 0,98 | | | 0,34 | | |  |
| C50 males | | | Slope 1 | | | -0,03 | | | | 0,03 | | -0,97 | | | 0,35 | | |  |
| C50 total | | | Intercept 1 | | | -1586,51 | | | | 413,85 | | -3,83 | | | 0,00 | | |  |
| C50 total | | | Slope 1 | | | 0,82* | | | | 0,21 | | 4,00 | | | 0,00 | | |  |

**Prostate Cancer**

**
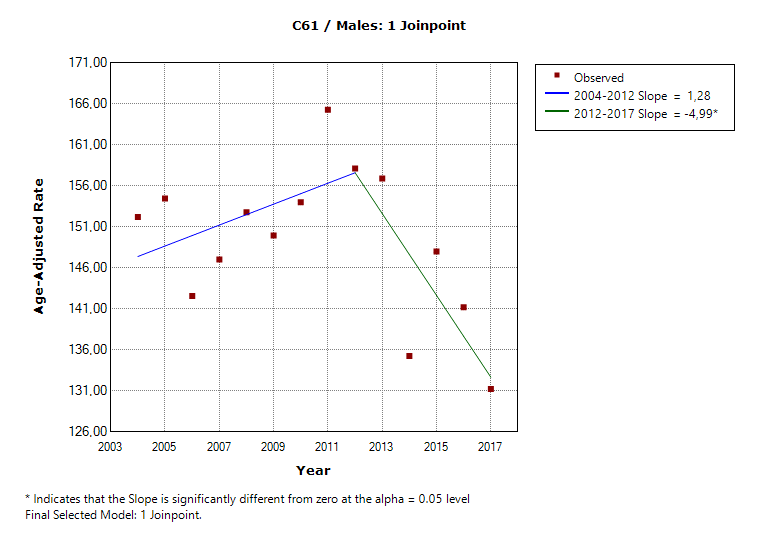
**

| **Model Statistics for Prostate Cancer** | | | | | | | | | | | | | | | |  |
| --- | --- | --- | --- | --- | --- | --- | --- | --- | --- | --- | --- | --- | --- | --- | --- | --- |
| **Cohort** | **Number of Joinpoints** | **Number of Observations** | | **Number of Parameters** | | **Degrees of Freedom** | | | **Sum of Squared Errors** | | **Mean Squared Error** | | **Autocorrelation**  **Parameter** | | |  |
| C61 | 1 | 14 | | 4 | | 10 | | | 8,71 | | 0,87 | | Uncorrelated | | |  |
| **Estimated Joinpoints** | | | | | | | | | | | | | | | | |
| **Cohort** | | | | | **Joinpoint** | | | **Estimate** | | | | **Lower CI** | | | **Upper CI** | |
| C61 | | | | | 1 | | | 2012 | | | | 2009 | | | 2014 | |
| **Estimated Regression Coefficients (Beta)** | | | | | | | | | | | | | | | | |
| **Standard Parameterization** | | | | | | | | | | | | | | | | |
| **Cohort** | | | **Parameter** | | | | **Param Estimate** | | | **Standard Error** | | | | **Test Statistic (t)** | **Prob > \|t\|** | |
| C61 | | | Intercept 1 | | | | -2410,28 | | | 2365,16 | | | | -1,02 | 0,33 | |
| C61 | | | Slope 1 | | | | 1,28 | | | 1,18 | | | | 1,08 | 0,31 | |
| C61 | | | Slope 2 - Slope 1 | | | | -6,26 | | | 2,34 | | | | -2,68 | 0,03 | |
| **General Parameterization** | | | | | | | | | | | | | | | | |
| **Cohort** | | | **Parameter** | | | | **Param Estimate** | | | **Standard Error** | | | | **Test Statistic (t)** | **Prob > \|t\|** | |
| C61 | | | Intercept 1 | | | | -2410,28 | | | 2365,16 | | | | -1,02 | 0,33 | |
| C61 | | | Intercept 2 | | | | 10189,26 | | | 4066,43 | | | | 2,51 | 0,03 | |
| C61 | | | Slope 1 | | | | 1,28 | | | 1,18 | | | | 1,08 | 0,31 | |
| C61 | | | Slope 2 | | | | -4,99* | | | 2,02 | | | | -2,47 | 0,04 | |

**
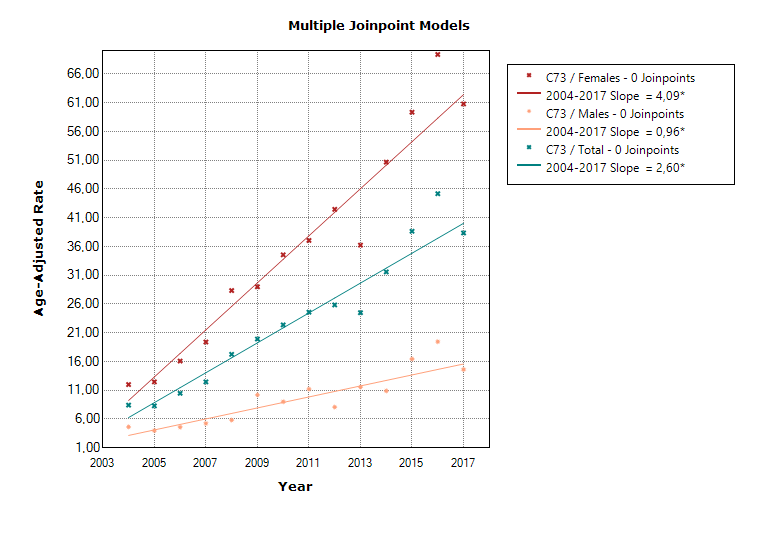
Thyroid Cancer**

| **Model Statistics** | | | | | | | | | | | | | | | |  |  |  |
| --- | --- | --- | --- | --- | --- | --- | --- | --- | --- | --- | --- | --- | --- | --- | --- | --- | --- | --- |
| **Cohort** | **Number of Joinpoints** | **Number of Observations** | | | **Number of Parameters** | | | **Degrees of Freedom** | **Sum of Squared Errors** | | | **Mean Squared Error** | | **Autocorrelation**  **Parameter** | |  |  |  |
| C73 females | 0 | 14 | | | 2 | | | 12 | 28,73 | | | 2,39 | | Uncorrelated | |  |  |  |
| C73 males | 0 | 14 | | | 2 | | | 12 | 18,23 | | | 1,52 | | Uncorrelated | |  |  |  |
| C73 total | 0 | 14 | | | 2 | | | 12 | 33,64 | | | 2,80 | | Uncorrelated | |  |  |  |
| **Estimated Regression Coefficients (Beta)** | | | | | | | | | | | | | | | | | | |
| **Standard Parameterization** | | | | | | | | | | | | | | | | | | |
| **Cohort** | | | **Parameter** | | | **Param Estimate** | | | | **Standard Error** | | | | | | **Test Statistic (t)** | **Prob > \|t\|** | |
| C73 females | | | Intercept 1 | | | -8191,47 | | | | 535,90 | | | | | | -15,29 | 0,00 | |
| C73 females | | | Slope 1 | | | 4,09 | | | | 0,27 | | | | | | 15,34 | 0,00 | |
| C73 males | | | Intercept 1 | | | -1912,61 | | | | 242,19 | | | | | | -7,90 | 0,00 | |
| C73 males | | | Slope 1 | | | 0,96 | | | | 0,12 | | | | | | 7,93 | 0,00 | |
| C73 total | | | Intercept 1 | | | -5202,66 | | | | 339,74 | | | | | | -15,31 | 0,00 | |
| C73 total | | | Slope 1 | | | 2,60 | | | | 0,17 | | | | | | 15,37 | 0,00 | |
| **General Parameterization** | | | | | | | | | | | | | | | | | |  |
| **Cohort** | | | | **Parameter** | | | **Param Estimate** | | | | **Standard Error** | | **Test Statistic (t)** | | **Prob > \|t\|** | | |  |
| C73 females | | | | Intercept 1 | | | -8191,47 | | | | 535,90 | | -15,29 | | 0,00 | | |  |
| C73 females | | | | Slope 1 | | | 4,09* | | | | 0,27 | | 15,34 | | 0,00 | | |  |
| C73 males | | | | Intercept 1 | | | -1912,61 | | | | 242,19 | | -7,90 | | 0,00 | | |  |
| C73 males | | | | Slope 1 | | | 0,96* | | | | 0,12 | | 7,93 | | 0,00 | | |  |
| C73 total | | | | Intercept 1 | | | -5202,66 | | | | 339,74 | | -15,31 | | 0,00 | | |  |
| C73 total | | | | Slope 1 | | | 2,60* | | | | 0,17 | | 15,37 | | 0,00 | | |  |
